# Diversity and ethnomycological importance of mushrooms from Western Himalayas, Kashmir

**DOI:** 10.1186/s13002-022-00527-7

**Published:** 2022-04-13

**Authors:** Tariq Saiff Ullah, Syeda Sadiqa Firdous, Wayne Thomas Shier, Javeed Hussain, Hamayun Shaheen, Muhammad Usman, Maryam Akram, Abdul Nasir Khalid

**Affiliations:** 1 Department of Botany, University of Kotli, Azad Jammu and Kashmir, Kotli, 11100 Pakistan; 2 Department of Botany, University of Azad Jammu and Kashmir (UAJK), Muzaffarabad, 13100 Pakistan; 3 Department of Botany, Women University of Azad Jammu and Kashmir, Bagh, 12500 Pakistan; 4 Department of Medicinal Chemistry, College of Pharmacy, University of Minnesota, 308 Harvard St., SE Minneapolis, MN 55455 USA; 5 Fungal Biology and Systematics Research Laboratory, Institute of Botany, University of Punjab, Quaid-e-Azam Campus 54590, Lahore, Pakistan

**Keywords:** Ethnomycology, Diversity of mushrooms, *Laetiporus sulphureus*, Morels, Traditional uses of mushrooms

## Abstract

**Background:**

Wild edible mushrooms (WEM) are economically significant and used in traditional medicines worldwide. The region of Jammu and Kashmir (Western Himalayas) is enriched with the diversity of edible mushrooms, collected by the rural people for food and income generation. This is the first detailed study on diversity and ethno-medicinal uses of mushrooms from the State of Jammu and Kashmir.

**Methods:**

Consecutive surveys were conducted to record ethnomycological diversity and socio-economic importance of wild edible mushrooms value chain in rural areas of Azad Jammu and Kashmir during 2015–2019. Ethnomycological data were collected with a semi-structured questionnaire having a set of questions on indigenous mycological knowledge and collection and retailing of wild edible mushrooms. A total of 923 informants from the study area provided the results identifying the gender, type of mushroom species, medicinal uses, and marketing of mushrooms. Diversity of mushrooms was studied by using quadrat and transect methods. Principal component analysis (PCA) and detrended correspondence analysis (DCA) were also applied to the dataset to analyse the relationship between species distribution, the underlying environmental factors, and habitat types. PCA identified the major species-specific to the sites and put them close to the sites of distribution.

**Results:**

A total of 131 mushroom species were collected and identified during 2015–2019 from the study area. Ninety-seven species of mushrooms were reported new to the State of Azad Jammu and Kashmir. The dominant mushroom family was Russulaceae with 23 species followed by Agaricaceae, 16 species. Major mushroom species identified and grouped by the PCA were *Coprinus comatus, Lactarius sanguifluus, Amanita fulva, Armillaria gallica, Lycoperdon perlatum, Lycoperdon pyriforme, and Russula creminicolor*. *Sparassis crispa, Pleurotus* sp, and *Laetiporus sulphureus* were recorded most edible and medicinally significant fungi. Morels were also expensive and medicinally important among all harvested macro-fungal species. These were reported to use against common ailments and various health problems.

**Conclusions:**

Collection and retailing of WEM contribute to improving the socio-economic status, providing alternative employment and food security to rural people of the area. These mushrooms are used as a source of food and traditional medicines among the rural informants and could be used as a potential source of antibacterial and anticancer drugs in the future.

## Background

Mushrooms are fruiting bodies with distinctive carpophores of Basidiomycetes and some Ascomycetes [1]. They grow in the wild and are cultivated for food and medicines worldwide [2]. Diversity of ectomycorrhizal fungi studied from Pakistan revealed 23 species from eleven genera. Dominant mushrooms species were recorded from the genus *Hymenoscyphus* and *Inocybe* [3]. Fugal species have been identified using morphological and molecular techniques, used for food and culinary purpose [4]. Diversity studies of fungi have been carried out previously by [3–5] using standard methods. Targeted surveys for mushrooms species were found more efficient than random surveys [6]. Baseline fungal community data were obtained through plot-based diversity methods [7]. The quadrat method was also used to record fungal diversity and distribution [8]. The line transect method is also helpful to compare different fungal communities with each other and species conservation [9] and to gain prudence about the factors influencing the composition and association of fungal communities [10]. It also gives temporal variation in fungal growth and maturation [11].

Mushrooms have many health-promoting benefits and applications in traditional medicines [12–14]. Ethnomycology is a new area of research focused on the interaction of fungi with local communities. It includes cultural, recreational, and traditional uses of mushrooms [15, 16]. It is a naturally renewable and under-exploited resource contributing to improving rural livelihood [17]. Due to diverse ecological, medicinal, nutritional, and health-promoting properties, mushrooms are gaining prime importance among scientific and research communities throughout the world [18]. Wild mushrooms are non-timber forest products (NTFPs) collected as a source of food and income [19–21]. Collection and utilization of wild edible mushrooms (WEM) vary among the different communities [22]. These are collected and marketed for food and commercial values [23]. Folk taxonomic-based study of fungi is important because many species of fungi are going to extinct [24]. Traditional mycological knowledge is useful and transferred from one generation to other to safeguard the utilization and applications of edible mushrooms [25].

Morels are also a valuable source of food and income among the rural people of Pakistan [26]. These are used in traditional medicines against common ailments [27]. It is essential to transfer the folk knowledge of mushrooms among ethnic mountain communities to enhance the collection, utilization, and conservation of mushrooms [28].

The whole region of Azad Jammu and Kashmir (AJK) is blessed with diverse geographic and climatic conditions with a diversity of mushrooms. Despite a large number of ethnic groups in the state of Jammu and Kashmir, the ethnomycological data are poorly documented from the area and no comprehensive studies have been taken previously to explore such resources for human welfare. There is a lack of proper documentation on the diversity, specific habitat, ethnomycological uses, production, harvesting, and export of mushrooms. Present research work is designed to record species diversity of mushrooms in AJK, ethnomycological uses, and their commercial and economic importance.

## Methods

### Study area

The study area lies in the Western Himalayan regions of Azad Jammu and Kashmir between 32°-17′ and 36°-58′ North latitude and 73°-6′ and 80°-30′ longitude in the western part of the Indian subcontinent with an area of 13,297 square kilometres. The elevation from sea level ranges from three hundred and sixty meters in the south to 6325 m in the north. Average annual rainfall 1300 mm. The population is 4 million and the ratio between rural to urban populations is 88:12. Forestry, livestock, and agriculture are major economic activities for rural income. The climate of the study area is subtropical monsoon type in the lower range to moist temperate in the middle and subalpine to alpine in upper regions. The summer is hot at lower altitudinal zones and pleasant in upper zones with very cold winters. The area above 1200 m altitude receives heavy snowfall from November to April. The average temperature recorded in summer remains 34 to 25 °C and in winters, 10 to 4 °C. Annual rainfall (average) in the monsoon region is 900–1300 mm and in monsoon-free region it remains 35–140 mm [29].

### Data collection

Consecutive field visits were carried out to selected villages, local markets, shops of the study area for gathering information about mushroom collection, and selling. A semi-structured questionnaire (Appendix 1) was used to collect the information on the wild edible mushrooms value chain, hunting, collection, preservation, and retailing [30]. Primary and secondary information was collected from all the available resources. Primary information was gathered by structured and semi-structured interviews with collectors, consumers, and sellers. Secondary information was collected from different literature, thesis, maps, and websites. Both formal and informal discussions with forestry professionals, key informants, village elders, farmers, women, schoolteachers, social workers, and shopkeepers were carried out to identify and verify the facts. Information on edibility, medicinal uses, preservation methods, and any other uses was also recorded.

**Fig. 1:**
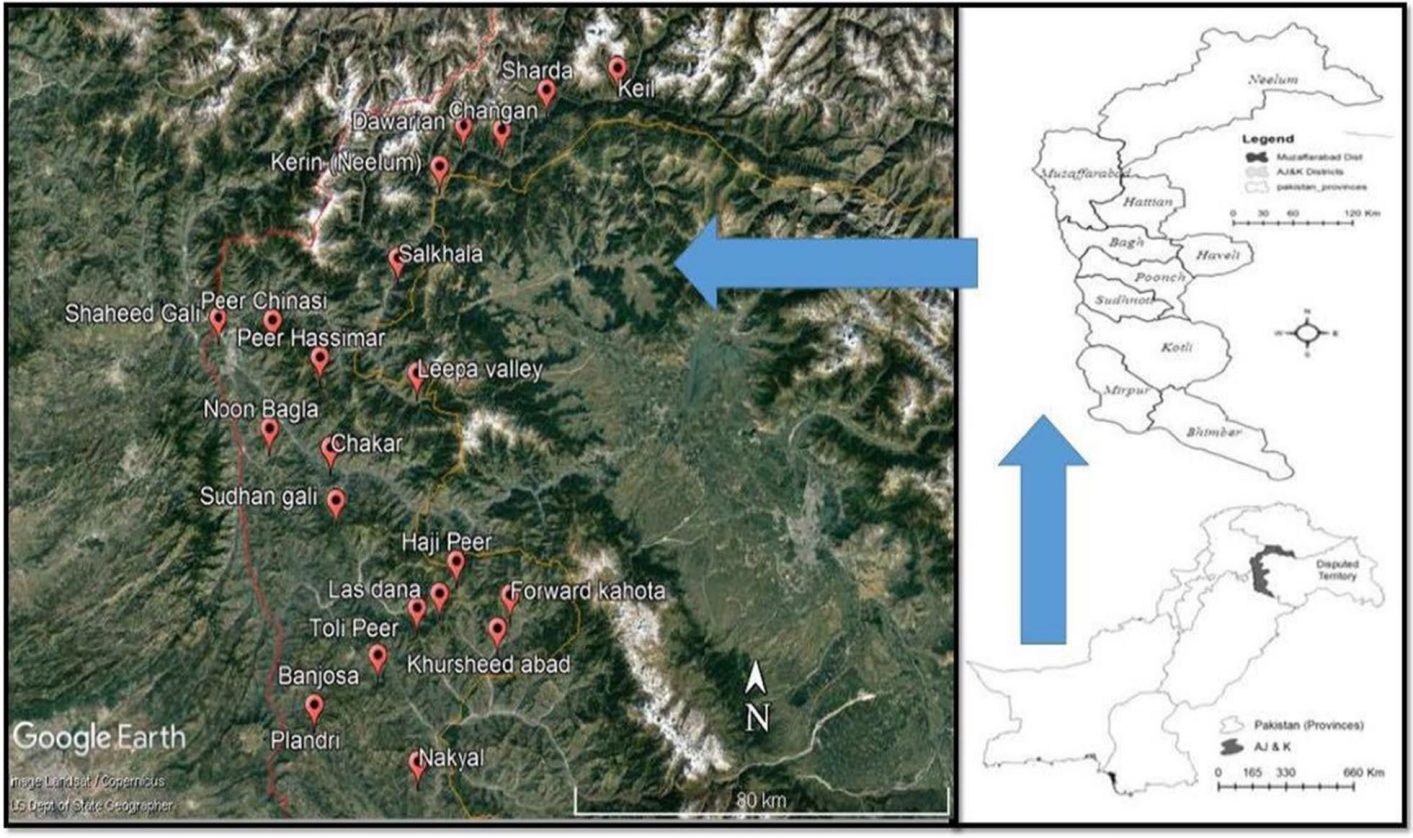
Map of the study area (right) and sampling sites (left)

All the major terrestrial ecological sites and hotspots for mushroom species from the state of Azad Jammu and Kashmir were selected for this study. Sampling sites were finalized through consecutive field visits based on specific geographic and ecological significance from representative vegetation zones of Azad Jammu and Kashmir. A total of 22 sites were selected from Neelum, Muzaffarabad, Hattian, Bagh, Heveli, Poonch, and Kotli districts of Azad Jammu and Kashmir during 2015–2019 to study mushroom diversity (Fig. 1 & Table 1).

**Table 1:** Different study sites and coordinates

No.	Site name	District	N	E	Elevation (m)
1	Peer Chinasi	Muzaffarabad	34°23′2.41	73°33′33.67	2596
2	Shaheed Gali	Muzaffarabad	34°23′1.01	73°25′16.55	1346
3	Peer Hassimar	Muzaffarabad	34°92′4.58	73°37′00.42	1901
4	Haji Peer	Bagh	33°58′2.61	74°04′40.43	2261
5	Las Dana	Bagh	33°55 ′2.54	73° 57′06.81	2331
6	Sudhan Gali	Bagh	34°44′6.34	73°44′11.74	2307
7	Banjosa	Poonch	33°48′2.75	73°49′25.92	1910
8	Toolipir	Poonch	33°53′4.72″	73°54′34.00	2334
9	Noon Bangla	Hattian	34°07′1.06″	73°40′11.50	2023
10	Chakar	Hattian	34°15′5.96″	73°37′01.85	1567
11	Palandri	Sudhnoti	33°43′3.37″	73°38′10.43	1517
12	Salkhala	Neelum	34°33′0.56″	73°53′14.53	1859
13	Dawarian	Neelum	34°44′0.53″	74°02′26.60	2431
14	Surgon	Neelum	34°47′5.80″	74°11′38.28	1921
15	Changan	Neelum	34°43′10.56″	74° 4′20.66	1920
16	Sharda	Neelum	34°46′5.36″	74°11′52.35	2475
17	Keil	Neelum	34°48′3.44″	74°21′25.70	2425
18	Forward Kahota	Haveli	33°54′1.58″	74°04′13.97	1883
19	Khursheed Abad	Havali	33°54′9.40″	74°12′21.59	2426
20	Nakyeal	Kotli	33°29′9.72″	74° 6′55.53″	1649
21	Leepa Valley	Hattian	34°18′5.25″	73°54′50.69″	2373
22	Kerin (Nagdar Valley)	Neelum	34°44′0.″76	74°02′26.00	2471

### Diversity of wild mushrooms

Sporophores of fungi were collected from forest communities of *Cedrus deodara* and *Pinus wallichiana*. For the documentation of fungal diversity quadrate and transect methods were used following standard protocols [7, 31–33]. The collection of samples was mostly carried out by targeted surveys to record a maximum number of mushroom species as described by [34]. Density, frequency, and relative values were calculated for the application of diversity indices [35]. Shannon diversity index was also calculated [36].

### Identification and preservation of Sporophores

A specific collection number was assigned to each sample in triplicate. Specific characters of habitat and associated plant species were also recorded. Sporophores were cleaned gently, soil particles were removed, and photographs were taken with a digital camera Nikon D5600. Fruiting bodies were left into the air for drying before packing for preservation. For easy drying, the larger Sporophores were cut down into many smaller pieces. Dried samples were packed and labelled with separate tag numbers for further analysis and future references. Specimens were finally cross-checked with the published material. The appropriate taxonomic literature was used for the proper identification of mushrooms [37–41]. Further citations were checked on MycoBank http://www.mycobank.org [42] and the index Fungorum database (http://www.indexfungorum.org/names/names.asp [43]. Final identification was made from fungal biology and systematic research laboratory Department of the Botany University of the Punjab Lahore. Specimen’s number were assigned to each sample and freeze at a temperature of − 80° for further future analysis.

## Results and discussion

### Diversity of mushrooms

**Fig. 2:**
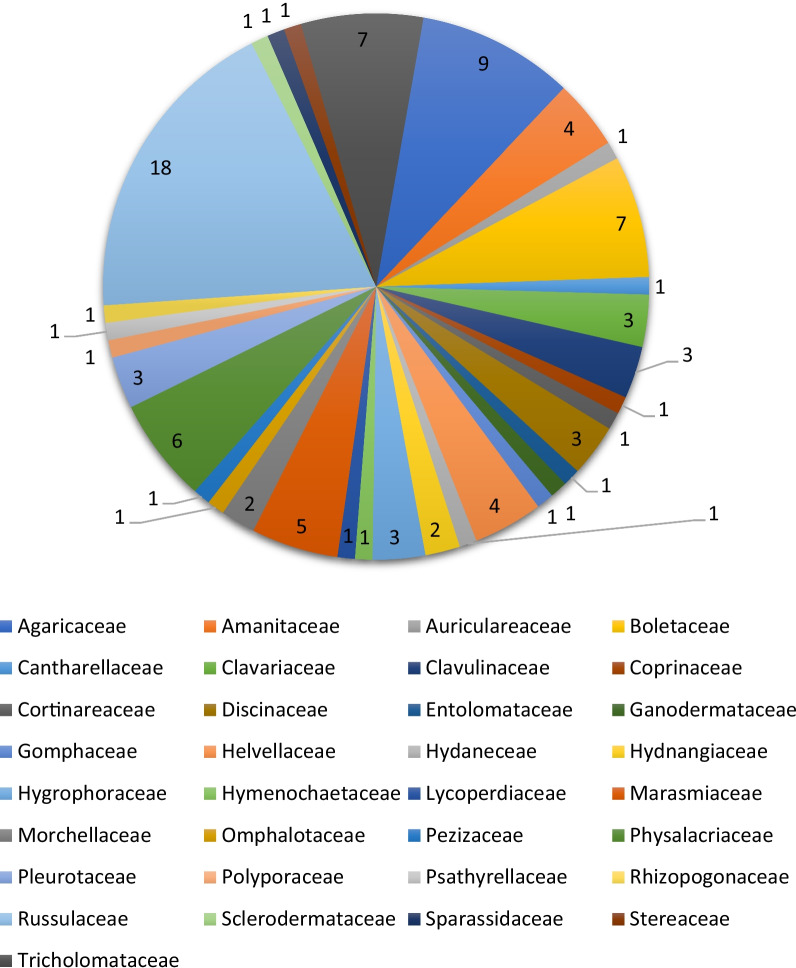
Mushrooms reported new to study area AJK

A total of 131 mushroom species were collected and identified up to species level during the study (Table 3) using standard methods [3–5]. Out of 131 mushroom species, 97 species of mushrooms were recorded new to the state of Azad Jammu and Kashmir (Fig. 2); however, few of these species have been identified from different parts of Pakistan at the molecular level previously [44]. Already identified mushroom species were morphologically cross-checked with published material. The dominant mushroom family was Russulaceae with 23 species followed by Agaricaceae, 16 species, Boletaceae, 10 species, Helvellaceae, 7 species, Tricholomataceae, and Physalaeriaceae 6 species were recorded in present investigations. Amanitaceae, Hymenochaetaceae, and Pleurotaceae were identified with five species each. *Russula* and *Lactarius* were the dominant genera. Only a few species of these genera were edible, and the maximum number of sporocarps decays on substratum after maturity. Inedible species were often collected for wound healing and other medicinal purposes. Most of the mushroom species growing naturally were collected by the rural for food and medicinal purposes. The maximum diversity of fungi was calculated in the Neelum Valley followed by Las Dana, Chakar, Noon Bangla, and Leepa in Jhelum Valley. These sites have maximum forest cover and diverse ecological conditions. The Basidiomycetes constituted the major proportion, i.e. 115 species, while Ascomycetes constituted 16 species. The majority of mushrooms collected belong to gilled fungi. Species of *Coprinus, Flammulina, Peziza, Armillaria,* and *Morchella* were found in clusters while other species occur in scattered patches. In Previous studies, six species of *Agaricus* were reported from Rawalakot, Azad Kashmir by [45]. Similarly [45] collected and described edible mushrooms, viz. *Armillaria mellea, Cantharellus cibarius, Craterellus cornucopioides, Flammulina velutipes*, and *Macrolepiota procera* from the area. Furthermore, more they added, *Amanita elliptica, A. muscaria var. alba, Ramaria aurea R. botrytis, Phallus impudicus, Morchella elata*, and *M. semilibera*, *Amanita ceciliae, A. subglobosa, A. pantherina, A. pachycolea, A. virosa, Volvariella bombycina*, and *V. speciosa* to Kashmir [46, 47] also contributed to the mushroom flora of AJK. They reported 25 edible mushrooms from different sites of the Azad Jammu and Kashmir. Dominant species of fungi collected during this study were also common with the previous studies [48–50]. These mushroom species grow during early spring in April to July in most of the studied areas. This pattern of diversity and distribution of fungal species associated with coniferous forest type was studied [51]. They reported *Russula* and *Lactarius* as a dominant genus associated with Himalayan cedar. Other studies on diversity of mushrooms in the literature revealed that most of the fungal communities were composed of Basidiomycetes [52]. Diversity and community stabilization of mushrooms depends upon different ecological factors including precipitation, soil organic matter and type of specific plant community. The sites which have some common geographic features also have similar species composition. This might be due to maximum annual rainfall and enough soil organic matter that promote the diversity of mushrooms because mushrooms grow maximum during the wet and rainy season in most parts of the world on different substrates [53]. Recently, fungal biology and systematics Laboratory University of Punjab is working on establishing Mycoflora data base and added many species to Mycota of Pakistan [54, 55].

**Table 2:** Demographic characteristics of Mushroom collectors in 6 districts of AJK (*N* = 923)

S. no.	Characteristics	Frequency	Percentage	Mean ± SEM
1.				
	Sex			
	Male	359	38.9	1.61 ± 0.01
	Female	564	61.1	
2.	Age group			
	< 18	163	17.6	2.80 ± 0.41
	19–30	238	25.8	
	31–40	259	28.1	
	41–50	140	15.2	
	> 50	123	13.3	
3.	Education level			
	Illiterate	157	17.0	2.88 ± 0.06
	Primary	238	25.8	
	Middle	210	22.8	
	Secondary	193	20.9	
	HS above	125	13.5	
4.	Employment status			
	Govt. servant	116	12.6	2.41 ± 0.26
	Farmer	366	39.7	
	Housewife	379	41.0	
	Retired	62	6.7	

### Principal component analysis

**Fig. 3:**
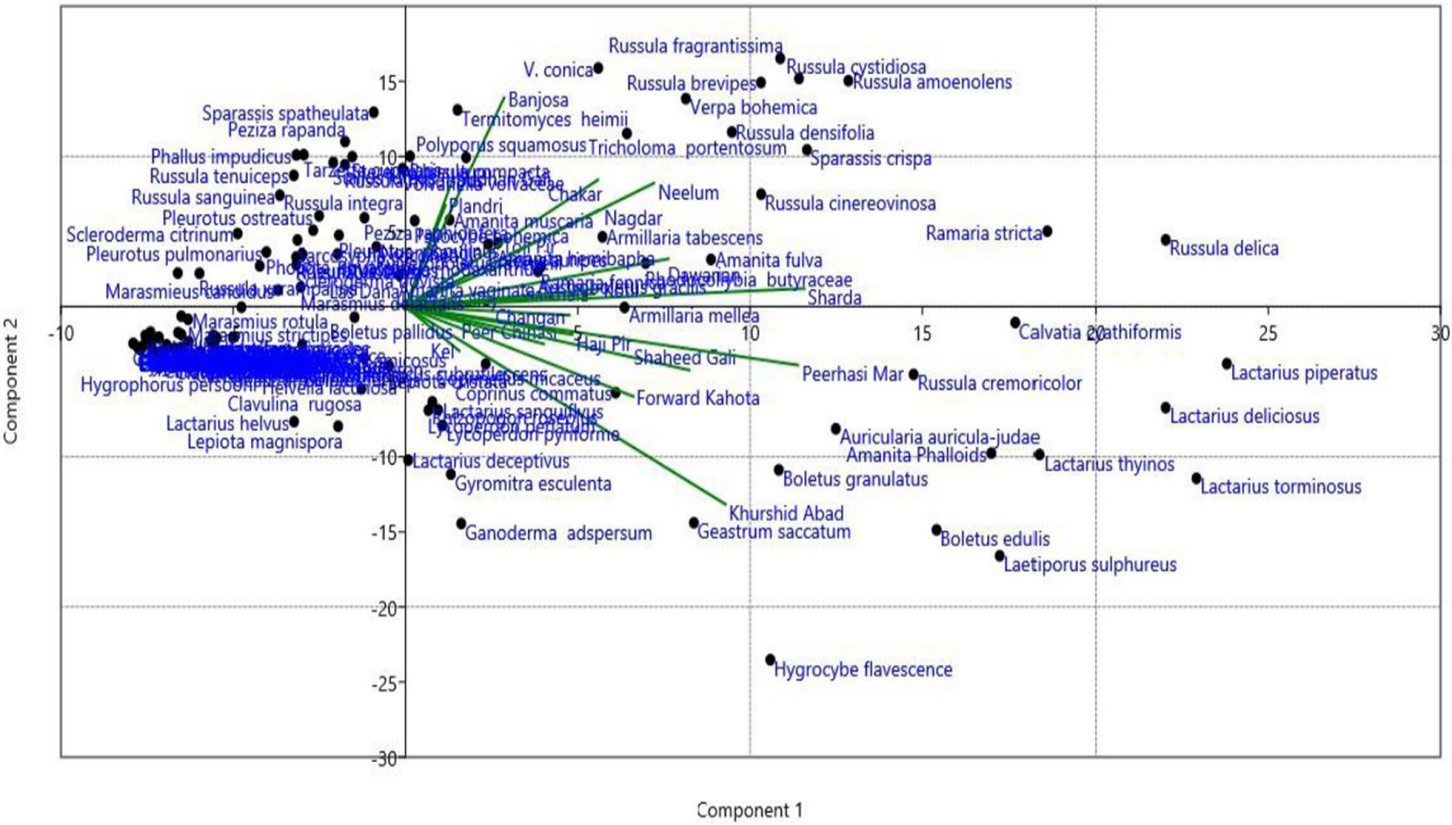
Expression of principal component analysis

PCA is used to determine and analyse the relationship between species distribution and the underlying environmental factors and habitat types. It is an advanced technique that maximizes the species scores concerning sampling sites having linear and appropriate weights. PCA identified the major species-specific to the sites and put them close to the sites of distribution. The sites grouped by the PCA based upon their species interrelationship are Peer Chinasi, Haji Peer and Peer Hasimar, Toolipeer, and Leepa. All these sites have little variations in the biotic factors including species composition and topography. These sites have some common geographic features which are responsible for similar species composition. Major mushroom species collected from these sites and grouped by the PCA are *Coprinus comatus, Lactarius sanguifluus, Amanita fulva, Armillaria gallica, Lycoperdon perlatum, Lycoperdon pyriforme, and Russula creminicolor*, these sites have shown a little correlation with a village Khawaja bandi kahuta Havali. The mushroom species grouped by the PCA are the common fungi that are present in these sites. On the other hand, Nagdar (Upper Neelum), Dawarian, Sharda, Taobut, Chakar (Noonbangla), Sudhan Gali, and Banjosa are grouped near to each other. These sites are almost lying in the temperate forest of AJK and have same topography, Forest cover, and precipitation pattern so their mushroom composition is nearly like each other. Major fungal species of these sites were *Amanita muscaria*, *Lactarius deliciosus*, *Gyromitra esculenta*, *Armillaria* sp, *Agaricus campestris*, *Russula brevipes, Polyporus squamosus, Trametes versicolor*, and *Laccaria* sp. Other mushroom species grouped at the centre of the PCA axis showed equal distribution and association with all the sites of the study area. These species have no specific distribution pattern. PCA identified five major keystone species from the data matrix and separated them along X-axis. *Lactarius piperatus, L. deliciosus, L. torminosus, Hygrocybe flavescens, and Russula delica* were extracted as most significant vectors having maximum Eigenvalue scores represented by their distinct placement on PCA biplot. These five species were characterized by the higher IVI values in the species dataset and enjoyed abundance and broad distribution across the study area. The major bulk of the fungal elements were clustered in the centre of the PCA biplot showing their random distribution without specific site or habitat preference. These species are most common and grow almost equally in different geographic conditions with slight changes in their growth period and maturation (Fig. 3).

### Detrended correspondence analysis

**Fig. 4:**
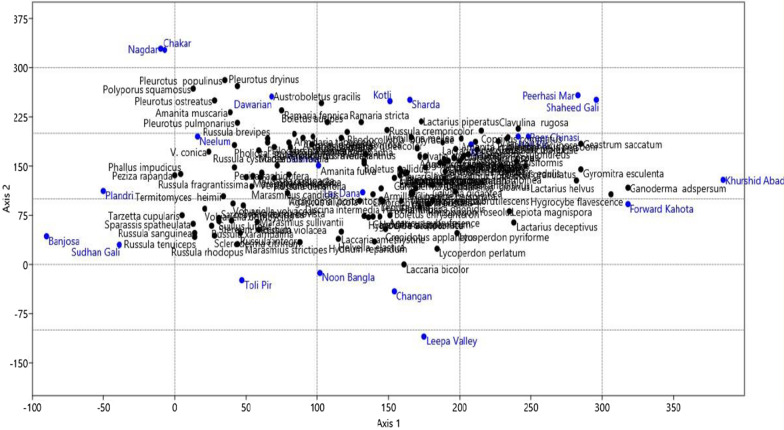
Expression of detrended correspondence analysis

**Fig. 5:**
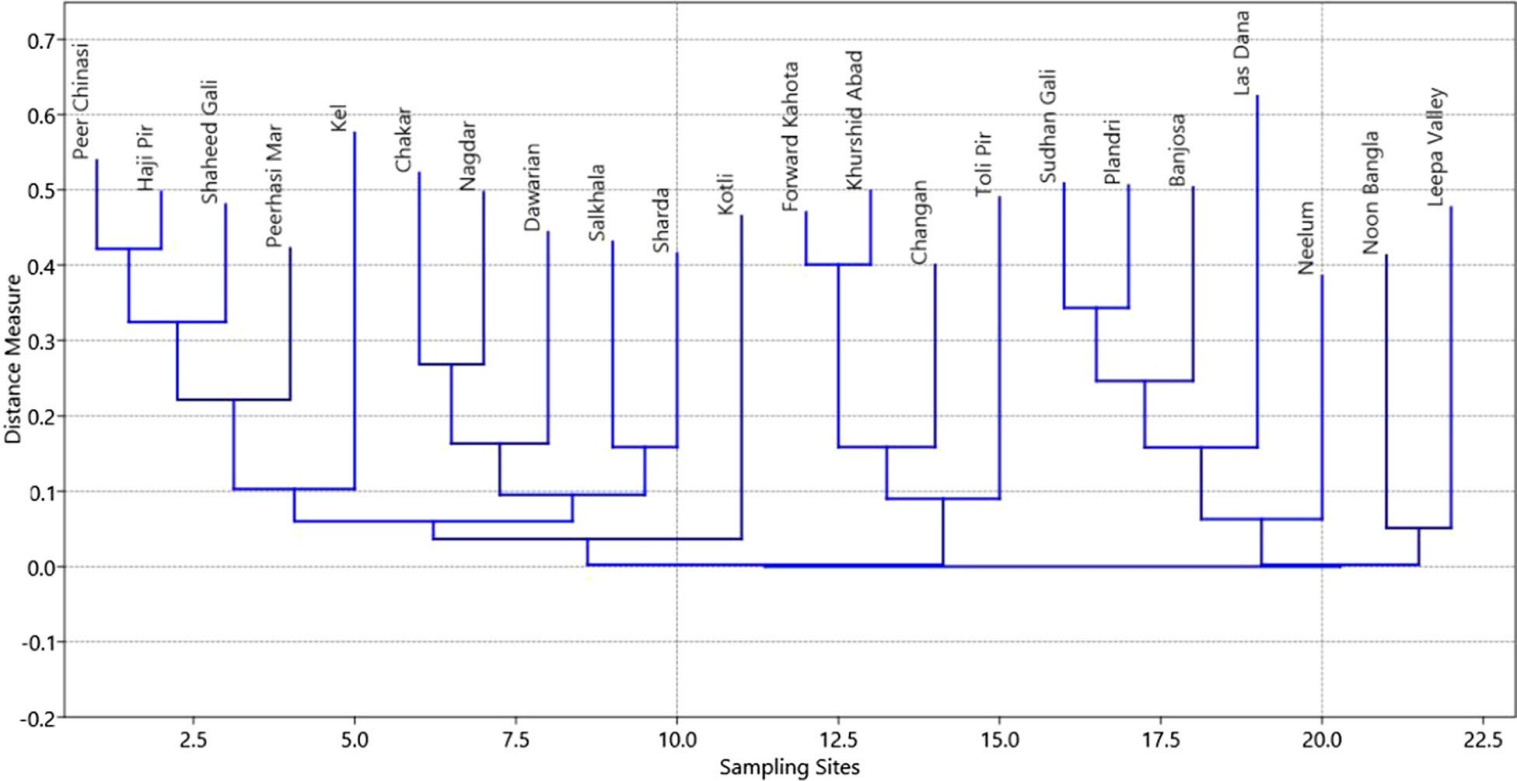
Expression of correspondence analysis among the different site

We subjected our species dataset to the DCA to extract the trends in species distribution and identify the specific habitat preference of the species represented by the sites. Our analysis results revealed uniform and continued species distribution patterns along specific environmental gradients with interpretable species-site assemblages. DCA separated the dataset into diffused but identifiable clusters. The Kotli site was separated at the top of X-axis with the characteristic species *Coprinellus micaceus.* This site lies in the subtropical zone with limited mushroom species growing during the monsoon*.* This specific microhabitat reflects the dominance of *Pinus roxburghii* and different grasses. Along the X-axis at the right side of the plot, different sites with similar species of mushrooms are grouped. These sites are Shaheed gali, Peer Chinasi, Sharda, Arangkeil, Noonbangla, Leepa Valley, Haji Peer, Dawarian, and Peer Hasimar. The Khurshidabad site in Havali was separated at the base of biplot and placed near to the Forward Kahuta with the characteristic mushroom species *Ganoderma lucidum* and *Hygrocybe flavescens*. Another identifiable cluster appeared at the left most of the biplot in the X-axis consisting of Chakar, Nagdar, and Upper Neelum placed with the Sharda site. While the left lowest groups are placed on the plot are the sites sharing the similar species composition these are Sudhan Gali, Banjosa, and Plandri (Figs. 4 and 5).

### Demographic characteristics and community involvement

Wild mushroom value chain is seen to be gender oriented dominated by women in collection (61.1%, *n* = 564) while men occupy only 38%, *n* = 359 out of the 923 respondents (Table 2). Women were found to participate in every mushroom activity such as collection to preservation while men contributed only to collection and selling. Similar findings were reported by [57] where female was found dominant in WEM collection. However, it was found that men dominated in selling of mushrooms (70%) to local shops, restaurant, markets, and local mushrooms entrepreneurs. The preponderance of female collectors in present study is supported by another research [58–60]. Every stage of mushroom activities from collection to processing and even marketing was led by women in this study. Poor involvement of men in mushroom activities might be due to the belief that mushroom collection is only art for remote areas of women. In remote areas of studied districts of AJK, women are mostly unemployed, dedicating themselves to household and subsistence activities. Mushroom collection and selling are one of their sources of food and income. The study revealed that collection activities are dominated by people of middle age (53.9%) especially those of 31–50 years old between the ages ranged 14–85, followed by 19–30 (25.8%), by 14 and over (17.6%), and by 50 and above (13.3%) (Table 2). Similar findings were also reported from the Finland [61] where it was shown that middle aged people by 30 (96.6%) or above involved in mushrooms collection activity. It revealed the participation of older, more experienced people in mushroom collection. Similar results on age distribution were also reported by [22]. Among 923 respondents, 25.8% had an education level of primary school, 22.8% middle school, 20.9% % secondary or high school, 17% illiterate, and 13.5% higher secondary, university, or colleges (Table 2). There were 41% housewives 39.7% farmers and entrepreneur, 12.6% employed, 6.7% retired from 923 respondents (Table 2). Data on education in the present study revealed that almost 83% of informants had a middle school education per the findings of [15] who indicated that mushroom collection or cultivation was mostly managed by less educated people in the rural areas.

### Socio-economic and ethnomycological importance of wild mushrooms

A total of 923 informants from 22 sites of selected districts were interviewed based on the harvesting, selling, and consumption of wild edible mushrooms. Mushrooms play a significant role in rural development. Many species of edible mushrooms and morels have been collected by the poor rural for a socio-economic purpose and rural livelihood in terms of economic development. Morels are collected by the people of rural areas of AJK for medicinal and commercial purposes. *Morchella conica, M. costata, M. esculanta, M. elata*, and *M. tridentina* were considered highly prized morel species. These morel species widely grow under the dense forest cover of *Pinus wallichiana* and *Cedrus deodara* in association with *Viburnum grandiflorum*. Among morels, *Morchella esculanta* and *M. tridentina* were valuable morels and considered good for export due to compact fruiting bodies, less moisture, and higher nutritional contents. *M. conica* has more water contents than the *M. esculanta* and turns dark black, which affects the preservation as well as its marketing. One kilogram of dried morel is solid in the market up to 32 thousand (Pakistani rupees) PKR. One kilogram of dry morels can fulfil the basic needs of a family of an average size. Prices of dried morels vary from market to market. In a village (Neelum) average price of 1 kg of dried morel is between 30,000 and 32,000 PKR. Other edible mushroom species *Pleurotus ostreatus* and *Agaricus campestris* were supplied to the famous hotels of the city. One Kg of dried mushroom is sold in 1500-2000PKR. These mushrooms are mostly used in dishes for foreign visitors. Mushrooms are collected worldwide as a source of food and income. Edible fungi, i.e. *Cantharellus cibarius*, *Lactarius deliciosus,* and *Russula* sp., were collected and sold in the market for food purposes [62]. More than 300 species of mushrooms were collected by different ethnic groups in Mexico for nutritional and medicinal purposes [63]. In China, local farmers earn up to 62% of their cash income through mushroom export [30]. Mushrooms play a significant role in rural development. Many species of edible mushrooms and morels have been collected by the rural for a socio-economic purpose [56, 64] and rural livelihood in terms of economic development [63]. Prices of dry mushrooms are higher than fresh mushrooms. Similarly, those mushrooms which are exported showed higher prices. The most common species collected and used for trade-in neighbouring countries of Pakistan are, for example, *Boletus* spp. *Lactarius* sp., *Suillus bovinus, Russula* sp., and *Termitomyces* sp. [46, 65]. In the present investigation, the socio-economic data showed that a family collects an average of 3–4 kg morels with an average income of about PKR 0.1-0.120 million in a season. Fifty-six species of mushrooms were reported as edible previously from Pakistan and unfortunately because of over-collection, urbanization, and deforestation some species are threatened [66].

Mushrooms are natural sources of bioactive compounds used in alternative traditional medicines. Today, in parallel with the increase in the number of diseases, alternative medicine, and their usage is also increasing. It might be due to the disadvantages or side effects of drugs. Mushrooms have compounds that decrease oxidative stress and improve health [67, 68]. Many unexplored species of medicinally and commercially important mushrooms were widely distributed in the forests of Azad Jammu and Kashmir. Mushroom species growing naturally were collected by the rural people for food and medicines. In previous studies, medicinally significant mushrooms from the Neelum Valley have been reported [27, 77]. They are also collected in different countries of the world like the UK, Sweden, France, and Mexico [62, 74]. In the present study, twenty-six species of mushrooms were recorded as medicinally important which are used for the treatment of some common ailments. Among these mushrooms *Fistulina* sp., *Hericium erinaceus*, *Laetiporus sulphureus, Polyporus squamosus, Ramaria fennica, Sparassis crispa, Morchella elata, M. conica, M. tridentina*, and *M. deliciosa* were the most delicious and widely used species as a nutritive food by the rural people of Neelum Valley and Jhelum Valley. *Morchella esculanta* is reported to contain antioxidant, anticancer, and anti-inflammatory properties and is used as delicious food [68]. Soup of dried fruiting bodies of *Ramaria fennica* is used by women during breastfeeding to improve lactation. *Ramaria fennica* and morel species were considered effective against common cough and cold. Many mushroom species are considered medicinally important and used against stomach problems, heart burning, and wound healing without considering any side effects or toxicity. Previously, it is reported that extract and powder of mushrooms are used in traditional medicines and have reported uses as a liver tonic, blood purifiers, fertility issue, and diabetes [69]. Fruiting bodies of *Laetiporus sulphureus* are dried into a fine powder and used with milk as a portion of healthy food and anti-seminal weakness. Previously, it is reported that *Laetiporus sulphureus* is used against speedy recovery of wounds and common cold [6]. In another study, it is found that dry powder of this mushroom is helpful to expel a retained placenta in women and against stomach pain [30]. Use values of mushrooms species recorded during the study are given in (Table 3). In the present study, we have found the use of morels in different traditional home remedies against common ailments, fever, cough, and cold. Soup of *Morchella* is considered nutritious and used to treat the common cold. Extract of many edible species of mushrooms is effective against different human diseases like coronary disorders, oxidative stress, and cancer and provides different physiological benefits to consumers [64]. *Sparassis crispa* and *Polyporus squamosus* were used to treat stomach issues and considered healthy food. Old villagers prefer to use these mushrooms as a source of food. People use *Morchella* species, *Hydnum repandum, Sparassis crispa*, and *Polyporus squamosus* against stomach problems, *Lycoperdon perlatum*, and *Auricularia auricula* in wound healing and as anti-hypertension agents. *Armillaria mellea*, *Boletus badius, Cantharellus cibarius, Pleurotus ostreatus*, and *Lactarius deliciosus* contain bioactive organic contents with reported uses in traditional medicines [70]. Sher and Shah [26] reported that morels were utilized both for food as well as medicines to cure different diseases.

**Table 3:** List of Mushrooms species with their Ethno-mycological uses

No.	Name of Species	Family	Edibility Status	Ethno-mycological uses	Ecology	Voucher specimen Number	Region	Reference
1	*Agaricus amicosus* Kerrigan.	Agaricaceae	Edible	Not used	Saprobic, scattered in fir litter	TS-106	Neelum AJK	Present study
2	*A. campestris* L.	Agaricaceae	Edible	Consumed as food	Saprobic, growing in a grassy area	TS-107	AJK	[49, 50]
3	*A. silvicolae-similis* Bohus & Locsmándi	Agaricaceae	Edible	Not consumed	Saprobic, growing on decomposed wood	TS-110	AJK	[49, 50]
4	*A. subrutilescens* (Kauffman) Hotson & D. E. Stuntz	Agaricaceae	Edible	Consumed as food	Saprobe, growing under coniferous forest	TS-109	AJK	Present study
5	*Amanita fulva* Fr	Amanitaceae	Inedible	Not consumed	Mycorrhizal with conifers or hardwoods	TS-110	AJK	Present study
6	*A. hemibapha* (Berk. &Broome) Sacc	Amanitaceae	Poisonous	Poisonous	Saprobic	TS-111	AJK	Present study
7	*A. muscaria* (L.) Lam	Amanitaceae	Poisonous	Poisonous	Mycorrhizal with pine and oak	TS-112	AJK	Present study
8	*A. phalloides* (Vaill. ex Fr.) Link	Amanitaceae	Deadly poisonous	Poisonous	Mycorrhizeal with oaks	TS-113	AJK	[75]
9	*A. vaginata* (Bull.) Lam	Amanitaceae	Edible	Not consumed as food	Mycorrhizeal with pines and oaks	TS-114	AJK	Present study
10	*Apioperdon pyriforme* (Schaeff.) Vizzini	Agaricaceae	Edible/medicinal	Consumed as food	Saprobic on deadwood of hardwoods or conifers	TS-115	Pak	[51]
11	*Armillaria gallica* Marxm. & Romagn	Physalacriaceae	Edible	Consumed as food	Saprophytic, on organic matter and soil	TS-120	AJK	Present study
12	*A. mellea* (Vahl) P. Kumm	Physalacriaceae	Edible	Consumed as food	Parasitic on the hardwoods, on conifers produce white rot in the wood	TS-121	Neelum AJK	Present study
13	*Auricularia auricula-judae* (Bull.) Quel	Auriculriaceae	Edible/medicinal	Used in weakness after childbirth, anti-hypertension	Grows in groves of trees, on logs and dead branches	TS-122	AJK/KPK	[52]
14	*A. gentilis* (Quél.) Pouzar	Boletaceae	Edible	Not consumed	Mycorrhizal with conifers	TS-123	AJK	Present study
15	*Boletus aureissimus* (Murrill) Singer	Boletaceae	Edible	Not consumed	Mycorrhizal with oaks	TS-124	AJK	Present study
16	*B. chrysenteroides* Snell	Boletaceae	Edible	Used as food	Mycorrhizal with oaks and conifers	TS-125	AJK	Present study
17	*B. edulis* Bull. Herb. Fr	Boletaceae	Edible	Used as food	Mycorrhizal with hardwoods	TS-126	AJK/KPK	[51, 52]
18	*Bovista utriformis* (Bull.) Fr	Agaricaceae	Edible	Consumed as food	Sandy ground	TS-127	AJK	Present study
19	*Coprinellus micaceus* (Bull.) Vilgalys, Hopple & Jacq. Johnson	Psathyrellaceae	Medicinal	Used in traditional medicines	Saprobic grow on decaying wood	TS-10	AJK	Present study
20	*Calvatia cyathiformis* (Bosc) Morgan	Agaricaceae	Edible	Consumed as food	Saprobic, grow in grass	SG-16	Kaghan Valley	Ahmed, 1950
21	*C. gigantea* (Batsch) Lloyd	Agaricaceae	Edible when young	Consumed as food	Saprobic, growing on grass, lawn, open places	SG-20	AJK	Present study
22	*Cantharellus cibarius* Fr	Cantharellaceae	Edible/medicinal	Consumed as food	Coniferous forest associated with moss	TS-003	Pakistan	[70]
23	*C. ignicolor* (R.H. Petersen) Dahlman	Cantharellaceae	Edible/medicinal	Consumed as food	Mycorrhizal with oaks, found in the cluster on mosses and grass	PC-132	AJK	Present study
24	*Chlorophyllum rhacodes* (Vittad.) Vellinga	Agaricaceae	Edible	Consumed as food	Saprobic, found in roadside, lawns, etc.	PC-133	AJK	[75, 76]
25	*C. olivieri* (Barla) Vellinga	Agaricaceae	Potentially dangerous	Consumed as food	Found in open areas	SG-134	AJK	Present study
26	*Clavaria fumosa* Pers	Clavariaceae	Edible	Consumed as food	Saprobic, found in a dense cluster in grass	TS-135	AJK	Present study
27	*Clavariadelphus ligula* (Schaeff.) Donk	Clavariaceae	Edible	Consumed as food	Saprobic, associated with fir needles on the ground	TS-138	AJK	Present study
28	*Desarmillaria tabescens* (Scop.) R.A. Koch & Aime	Physalacriaceae	Edible	Consumed as food	Saprobic on oaks	TS-139	AJK	Present study
29	*Clavulinopsis fusiformis* (Sowerby) Corner	Clavariaceae	Edible	Consumed as food	Saprobic, under hardwoods or conifers	TS-140	Neelum AJK	Present study
30	*Clavulina alta* Corner	Clavulinaceae	Edible	Consumed as food	Mycorrhizal with conifers	TS-141	Neelum AJK	Present study
31	*C. cinerea* (Bull.) J. Schrot	Clavulinaceae	Edible	Consumed as food	Mycorrhizal association with conifers	TS-142	Neelum AJK	Present study
32	*C. coralloides* (L.) J. Schröt	Clavulinaceae	Edible	Consumed as food	Mycorrhizal with conifers and hardwoods	SG-027	Neelum AJK	Present study
33	*Clitocybe acicula Singer*	Tricholomataceae	Edible	Not consumed	On debris of conifers	TS-143	AJK	Present study
34	*C. nebularis* (Batsch) P. Kumm	Tricholomataceae	Edible/uncommon/medicinal	Not consumed	Found under conifers	TS-76	AJK	Present study
35	*Clitopilus prunulus* (Scop.) P. Kumm	Entolomataceae	Edible	Not consumed	Saprobic, under, or conifers	PC-88	AJK	Present study
36	*Coprinus coffeicola* Massee, Bull	Hymenochaetaceae	Inedible	Inedible	Saprobic, under hardwoods	TS-144	AKJK	Present study
37	*C. commatus* (O. F. Mull.) Pers	Coprinaceae	Edible when young	Not consumed	Widely in grassland	TS-145	AJK	Present study
38	*Crepidotus applanatus* (Pres.) P. Kumm	Cortinareaceae	Edible	Not consumed	Under forest	TS-146	AJK	Present study
39	*Desarmillaria tabescens* (Scop.) R.A. Koch & Aime	Physalacriaceae	Edible	Consumed as food	Saprophytic on oaks	TS-150	AJK	Present study
40	*Exidia recisa (*Ditmar) Fr	Auriculareaceae	Inedible	Not consumed	Underwood and conifers	PC-89	Neelum AJK	Present study
41	*Floccularia luteovirens* (Alb. & Schwein.) Pouzar	Russulaceae	Edible	Not consumed	Ecto-Mycorrhizal, grow on the ground with pines	SG-19	AJK	Present study
42	*F. straminea* (P. Kumm.) Pouzar	Agaricaceae	Inedible	Not clear	Under confers	TS-151	AJK	Present study
43	*Flammulina fennae* Bas	Physalacriaceae	Edible	Not consumed	On older tree trunks and under conifers	TS-152	AJK	Present study
44	*F. ononidis* Arnolds	Physalacriaceae	Edible	Not consumed	On the ground and rotten trees	TS-153	AJK	Present study
45	*Fistulina* sp	Agaricomycetes	Edible/medicinal	Consumed as food	At the tree trunk of *Prunus padis*	TS-154	Neelum AJK	Present study
46	*Gyromitra bubakii* (Velen.) J. Moravec	Discinaceae	Edible on choice	Not consumed	Under forest	TS-155	AJK	Present study
47	*G. intermedia* (Benedix) Harmaja	Discinaceae	Edible on choice	Not consumed	Under forest	TS-156	AJK	Present study
48	*G. esculenta* (Pers.) Ex. Fr	Discinaceae	Conditionally edible /medicinal	Conditionally edible	Under Quercus trees	TS-157	AJK	[27]
49	*Ganoderma adspersum* (Schulzer) Donk	Ganodermataceae	Inedible/med	Not consumed	On the ground and rotten trees	TS-158	AJK	Present study
50	*G. lucidum* (Curtis) P. Karst	Ganodermataceae	Inedible/med	Medicinal	On the ground and rotten trees	TS-159	AJK	[45]
51	*G. applanatum* (Pers.) Pat	Ganodermataceae	Medicinal	Medicinal	Under Quercus trees	TS-160	AJK	[68]
52	*Geastrum saccatum* Fr	Geastraceae	Inedible	Not consumed	Under Quercus trees	TS-161	Pakistan	[69]
53	*G. pedicellatum* (Batsch) Dörfelt & Müll. Uri	Agaricaceae	Unknown	Not confirm	On grassy ground	TS-162	AJK	[50]
54	*G. triplex* Jungh	Geastraceae	Inedible	Not consumed	Under Quercus trees	SG-173	Pakistan	[50, 51]
55	*Helvella sulcata* Afzel	Helvellaceae	Edible	Consumed s food	On decaying hardwoods stumps	SG-174	AJK	Present study
56	*H. elastica* Bull	Helvellaceae	Inedible	Inedible	On the ground, on decaying wood	SG-175	AJK	Present study
57	*H. crispa* (Scop.) Fr	Helvellaceae	Edible	Consumed as food	Mycorrhizal. Growing under conifers or hardwoods	SG-176	Kaghan Valley	[69]
58	*H. lacunosa* Afzel	Helvellaceae	Conditionally edible/medicinal	Consumed as food	Not consumed	SG-177	Kaghan Valley	[69]
59	*H. fibrosa* (Wallr.) Korf	Helvellaceae	Edible	Not consumed	On confers or wood of hardwoods	SG-178	Pakistan	[69]
60	*Hohenbuehelia* sp. T-62 (LAH, 1193)	Pleurotaceae	Edible/medicinal	Consumed as food	Saprobic grows on decaying sticks and branches in damp spots on the forest floor	SG-179	Neelum AJK	Present study
61	*Hydnum repandum* L	Hydaneceae	Edible/medicinal	Consumed s food	Under Quercus trees	SG-180	AJK	Present study
62	*Hygrocybe acutoconica* (Clem.) Singer	Hygrophoraceae	Edible	Consumed s food	On confers or wood of hardwoods	SG-181	AJK	Present study
63	*H. flavescens* (Kauffman) Singer	Tricholomataceae	Inedible	Not consumed	On confers or wood of hardwoods	SG-182	AJK	Present study
64	*Hygrophorus piceae* Kuhner	Hygrophoraceae	Edible	Unknown	On confers or wood of hardwoods	SG-183	AJK	Present study
65	*H. persooni* Arnolds	Hygrophoraceae	Edible /medicinal	Unknown	On confers or wood of hardwoods	SG-184	AJK	Present study
66	*Imleria pallida* (Frost) A. Farid, A.R. Franck, & J. Bolin	Boletaceae	Unknown	Not consumed	Mycorrhizal with oaks	TS-185	AJK	Present study
67	*Laccaria amethystina* Cooke	Hydnangiaceae	Edible on choice/medicinal	Not consumed	Mycorrhizal with oaks	TS-186	AJK	Present study
68	*L. bicolor* Maire	Hydnangiaceae	Conditionally edible	Not consumed	Mycorrhizal with conifers, found in mosses	TS-187	AJK	Present study
69	*Lactarius deliciosus* (L.) Gray	Russulaceae	Edible/medicinal	Not consumed	Mycorrhizal with conifers	TS-188	Pak	[51]
70	*Lactarius sp*	Russulaceae	Edible	Consumed as food	grows under conifers on acidic soils	TS-189	AJK	Present study
71	*L. helvus* (Fr.) Fr	Russulaceae	Poisonous	Poisonous	Mycorrhizal with conifers	TS-190	AJK	Present study
72	*L. quieticolor* Romagn	Russulaceae	Edible	Not consumed	Mycorrhizal	TS-200	AJK	Present study
73	*L. torminosus* (Schaeff.) Pers	Russulaceae	Inedible	Inedible	Mycorrhizal, mixed forest	HP-007	AJK	Present study
75	*Lactifluus piperatus* (L.) Roussel	Russulaceae	Edible/medicinal	Inedible	On oak	SG-192	AJK	[50]
76	*Lepista ovispora* (J.E. Lange). Gulden	Tricholomataceae	Conditionally edible/med	Not consumed	Open grassland	SG-193	AJK	Present study
77	*Laetiporus sulphureus* Bull. Murrill	Fomitopsidaceae	Edible/medicinal	Consumed as food	On oak, prunus, Salix, etc.	TS-201	AJK	[51]
78	*Lepiota cristata*. (Bolton) P. kumm	Agaricaceae	Edible	Consumed as food	Saprobic, on forest, lawns, etc.	TS-202	Sohawa Shareef AJK	Present study
79	*L. magnispora* Murill	Agaricaceae	Inedible	Inedible	Saprobic, Found under hardwoods and conifers	TS-203	Neelum AJK	Present study
80	*Lepista luscina* (Fr.) Singer	Tricholomataceae	Edible	Not consumed	In mixed forest	TS-204	AJK	Present study
81	*L. irina* (Fr.) H.E. Bigelow	Tricholomataceae	Unknown	Not consumed	In mixed forest	TS-205	AJK	Present study
82	*Lycoperdon perlatum* Pers	Agaricaceae	Edible when young/medicinal	Consumed as food and wound healing	Open areas, grassy ground	TS-210	Pak	[69]
83	*Leucopaxillus giganteus* Calonge & M	Stereaceae	Inedible	Inedible	Saprobic on deadwood of oaks	TS-002	AJK	Present study
84	*Morchella tridentina* Bres	Morchallaceae	Edible/medicinal	Used in cough and cold, highly medicinal	Saprobic on deadwood or conifers	T-05 & T-06	AJK	Present study
85	*M. deliciosa* Fr	*Morchella*ceae	Edible/medicinal	Consumed as food and medicinal	On humus-rich soil	T-02	AJK	Present study
86	*M. costata* Pers	*Morchella*ceae	Edible/medicinal	Consumed as food and medicinal	On leaf litter	T-04	Pak	[72]
87	*M. conica* Pers	Morchallaceae	Edible/medicinal	Consumed as food and medicine	under grass and conifers	T-07	Pak	[72]
88	*M. esculenta* Pers	Morchallaceae	Edible/medicinal	Used in cough and cold, highly medicinal	Saprobic on deadwood of hardwoods or conifer	T-08	AJK	[69]
89	*M. elata* Fr	Morchallaceae	Edible/medicinal	Consumed as food and medicinal	On grasses	T-09	Pak	[72]
90	*Marasmius abrubtipes* Corner	Marasmiaceae	Inedible	Not used	On humus-rich soil	TS-65	AJK	Present study
91	*M. abundans* Corner	Marasmiaceae	Inedible	Not used	On leaf litter	TS-66	AJK	Present study
92	*M. rotula* (Scop.) Fr	Marasmiaceae	Inedible	Not used	Saprobic on deadwood, hardwoods of conifer	TS-68	AJK	Present study
93	*M. strictipes (*Peck.) Singer	Marasmiaceae	Inedible	Not confirmed	Saprobic on deadwood of hardwoods or conifer	TS-69	AJK	Present study
94	*M. acerinus* Peck	Marasmiaceae	Inedible	Not confirmed	On grasses	TS-70	AJK	Present study
95	*Pleurotus dryinus* (Pers.) P. Kumm	Pleurotaceae	Edible when young	Consumed as food and medicinal	Saprobic, growing on oaks	TS-72	AJK	present study
96	*P. ostreatus* (Jacq.) P. Kumm	Pleurotaceae	Edible	Consumed as food	Saprobic on wood	TS-65	AJK	[75]
97	*Pholiota brunnescens* A.H. Sm. & Hesler	Strophariaceae	Inedible	Not consumed	Saprobic on wood	TS-212	AJK	Present study
98	*Polyporus septosporous* P.K. Buchanan & Ryvarden	Polyporaceae	Edible/medicinal	Consumed as food	Saprobic on decaying hardwood logs, etc.	TS-213	AJK	Present study
99	*Ramaria fennica* (P. karst.) Ricken	Gomphaceae	Edible	Consumed as food	Mycorrhizal with hardwoods	TS-214	AJK	Present study
100	*R. barenthalensis* Franchi & M	Russulaceae	Edible	Not consumed	Mycorrhizal with trees and shrubs	TS-215	AJK	Present study
101	*R. stricta* (Pers.) Quel	Gomphaceae	Edible	Consumed as food	Mycorrhizal and Saprobic	TS-216	AJK	Present study
102	*Rhodocollybia butyracea* (Bull.) Lennox	Omphalotaceae	Inedible	Not consumed	Saprobic, decomposing the litter of conifers	TS-217	AJK	Present study
103	*Russula amoenolens* Romagn	Russulaceae	Conditionally edible	Not consumed	Mycorrhizal with hardwoods and conifers	TS-218	AJK	Present study
104	*R. brevipes* Peck	Russulaceae	Edible	Not consumed	Mycorrhizal with conifers	TS-219	Pakistan	[71]
105	*R.cinereovinosa* Fatto	Russulaceae	Inedible	Inedible	Mycorrhizal with conifers, fir	TS-220	AJK	Present study
106	*R. collina* Velen Frost	Russulaceae	Inedible	Inedible	Mycorrhizal with hardwoods and conifers	T-46	AJK	Present study
107	*R. cremoricolor* Earle	Russulaceae	Unknown	Not clear	Mycorrhizal, mixed forests	T-47	AJK	Present study
108	*R. cystidiosa* Murrill	Russulaceae	Unknown	Not clear	Mycorrhizal with oaks	T-48	AJK	Present study
109	*R. delica* Fr	Russulaceae	Edible	Consumed as food	Found under broadleaved and coniferous wood	T-49	AJK	Present study
110	*R. densifolia* Secr. ex Gillet	Russulaceae	Edible	Not consumed	Mycorrhizal with conifers	PS-34	AJK	Present study
111	*R. fragrantissima* Romagn	Russulaceae	Inedible	Inedible	Mycorrhizal with hardwoods and conifers	PS-35	AJK	Present study
112	*R. integra* (L). Fr	Russulaceae	Conditionally edible	Inedible	Mycorrhizal with hardwoods and conifers	ND-09	AJK	Present study
113	*R. acriuscula* Buyck	Russulaceae	Edible/med	Not consumed	Mycorrhizal with hardwoods and conifers	ND-10	AJK	Present study
114	*R. tenuiceps* Kauffman	Russulaceae	Inedible	Inedible	Mycorrhizal with oaks	ND-11	AJK	Present study
115	*R. violacea* Quel	Russulaceae	Edible	Not consumed	Mycorrhizal with hardwoods and conifers	ND-12	AJK	Present study
116	*Rhizopogon* roseolus (Corda)Th. Fr	Rhizopogonaceae	Medicinal	Consumed as food	Ectomycorrhizal fungus	ND-16	Bagh AJK	Present study
117	*Suillus granulatus* (L.) Roussel,	Boletaceae	Edible	Not consumed	Mycorrhizal with pines	ND-17	AJK	Present study
118	*S. luteus* (L.) Roussel	Suillaceae	Edible	Not consumed	Mycorrhizal with pines	ND-19	Pakistan	[68]
119	*Suillellus luridus* (Schaeff.) Murrill	Boletaceae	Conditionally Edible	Consumed as food	Mycorrhizal with pines and other hardwoods	ND-20	AJK	Present study
120	*Scleroderma bovista, Fr*	Sclerodermataceae	Inedible	Inedible	Saprobic on the ground, mycorrhizal with hardwoods	PHM-07	Kaghan Valley	[72]
121	*S. citrinum* Pers	Sclerodermataceae	medicinal/poisonous	Consumed as food	Attached to soil my mycelial cords	PHM-08	Bagh AJK	Present study
122	*Stromatinia rapulum* (Bull.) Boud	Pezizaceae	Conditionally edible	Not consumed	Saprobic on well-decayed logs	PHM-12	AJK	Present study
123	*Sparassis spathulata* (Schwein.) Fr	Sparassidaceae	Edible when young	Used as stomach tonic and food	Pathogenic and Saprobic	PHM-13	AJK	Present study
124	*S. crispa* (Wulfen) Fr	Sparassidaceae	Edible/medicinal	Consumed as food/medicinal	Pathogenic and saprobic	PHM-14	Pakistan	[70]
125	*Tricholoma portentosum* (Fr.) Quel	Tricholomataceae	Edible and medicinal	Consumed as food	On Coniferous woods and oaks	ND22	AJK	Present study
126	*Volvopluteus gloiocephalus* (DC.) Vizzini, Contu & Justo	Pleurotaceae	Edible	Consumed as food	Saprobic, growing aggregates in gardens, lawns, woodchips, etc.	ND-27	AJK/KPK	[72]
127	*Volvariella volvaceae* (Bull.) Singer	Pleurotaceae	Edible	Consumed as food	Saprobic, growing in woodchips	SG-07	AJK/KPK	[72]
128	*V. bombycina* (Schaeff.) Singer	Pleurotaceae	Edible	Consumed as food	Saprobic, growing in woodchips	CHK-02	AJK/KPK	[72]
129	*Verpa bohemica* (Krombh.) J. Schroet	Helvellaceae	Conditionally edible	Consumed as food	Mycorrhizal. Found under hardwoods and conifers in early spring	PC-01	Neelum AJK	Present study
130	*V. conica* (O.F. Müll.) Sw	Helvellaceae	Conditionally edible	Consumed as food	Mycorrhizal. Found under hardwoods and conifers in early spring	CHK-02	Neelum AJK	Present study
131	*Xerocomellus chrysenteron* (Bull.) Šutara	Boletaceae	Edible	Food	Mycorrhizal with oaks and conifers	CHK-03	AJK	Present study

Ethno-mycological uses of mushrooms vary from region to region and even among the communities of the same area [71]. In Poland, edible mushroom species are used as food and medicines. Folk taxonomy is very important to share the knowledge and use of these mushroom species. Extract of mushrooms can be used due to cosmeceutical and nutricosmetic ingredients to treat inflammatory skin disease and hyperpigmentation [72]. Aqueous Extracts of *Polyporus squamosus*, *Morchella* spp., and *Sparassis crispa* are considered more effective against common diseases of the stomach by the rural informants of Kashmir. As it is reported that mushrooms are effective against different diseases, but the chemical evaluation is very important before using an extract of mushroom species [73]. Mushrooms are used in culinary traditional medicines and sometimes cooked in oil [74]. It is concluded that mushrooms potentially can provide opportunities to rural communities to generate income for household development in rural areas of Azad Jammu and Kashmir. Mushroom collection can provide opportunities to the low-income areas to improve their living standards in terms of income generation and socio-economic development. It is very important to raise awareness among the local communities/mushroom collectors, about the importance of mushrooms as food and medicines. Mushrooms, if well addressed in society, are a potential source of traditional medicines, anti-cancer compounds, food, and nutrition security specifically in developing countries.

### Mushrooms edibility in the study area

**Fig. 6:**
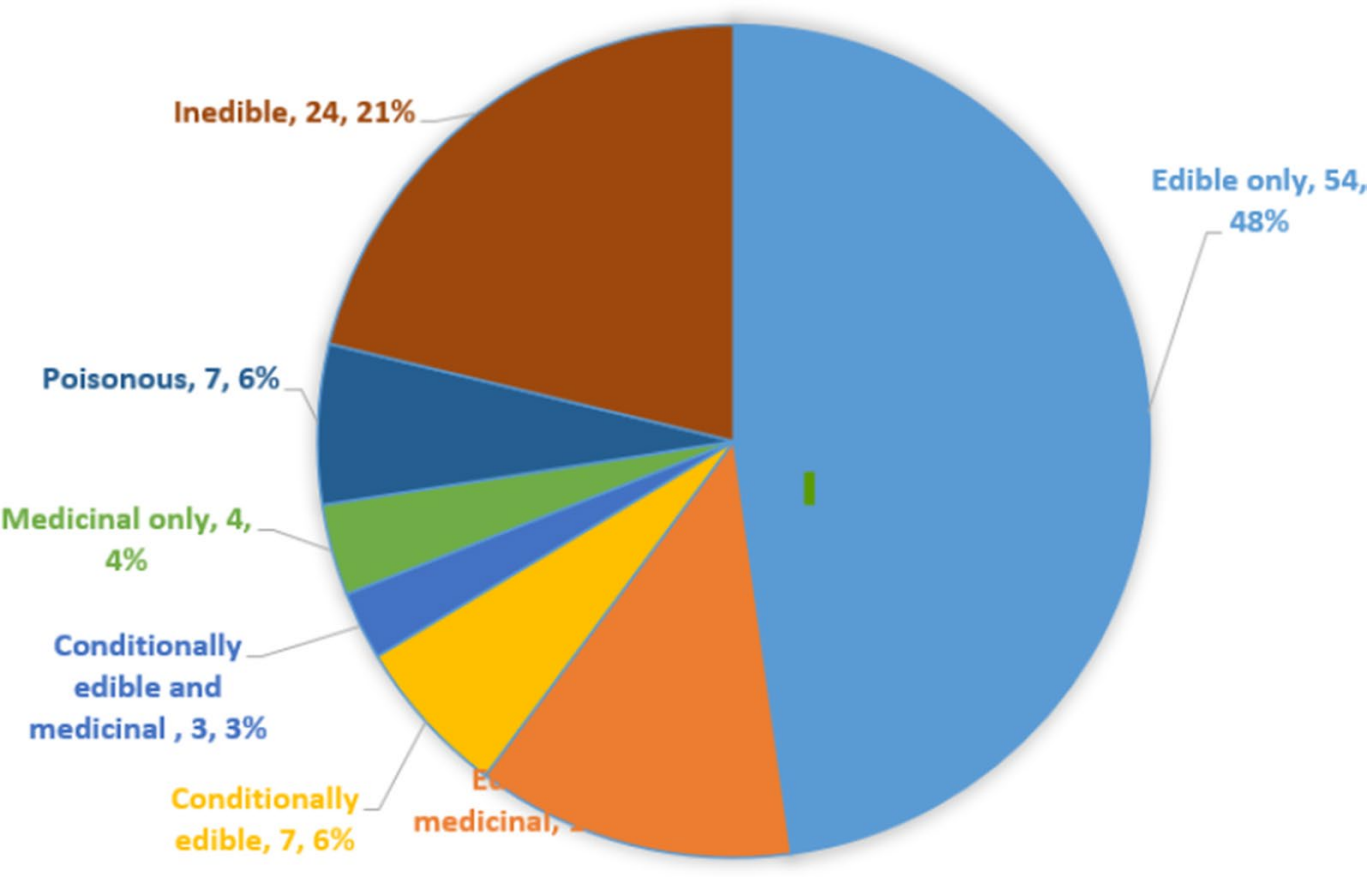
Category, number, and percentage use value of edible mushrooms of the study area

The state of Azad Jammu and Kashmir (AJK) is blessed with a fertile land, rich with diversity of mushrooms. Among the identified wild mushrooms, 54 (48%) were identified as edible, 24 (21%) inedible, 14 (12%) edible and medicinal (Fig. 6). *Lactarius deliciosus*, *Morchella* sp., *Pleurotus ostreatus*, *Polyporus squamosus Sparassis crispa,*, and *Laetiporus sulphureus* were collected by the rural people of the area as a source of food.  Edible mushrooms have been collected and consumed as food worldwide [4, 14, 27, 74]. Edible mushrooms like *Lactarius deliciosus* and *Ramaria* sp. have been collected and consumed in the neighbouring countries of Pakistan [78].

## Data Availability

Data sharing does not apply to this article as no datasets were generated or analysed during the current study.

## Appendix 1

See Table 4

**Table 4:** The questionnaire used for data collection from rural informants

S. no.	Information on mushroom	Respondent
i.	Who sells mushrooms, women or men?	–
ii.	Age of the vendors (five age groups): < 18, 19–30, 31–40, 41–50, > 50	–
iii.	The level of education (Illiterate, primary, middle, secondary, higher secondary and above)?	–
iv.	Employment status (Govt. servant farmer and entrepreneur, housewife, and retired)?	–
v.	Types of socio-economic data Wild or cultivated edible mushroom species local people know?	–
vi.	Which edible mushrooms have you collected?	–
vii.	Which mushroom species have you sold?	–
viii.	Which mushroom species have you used but not sold?	–
ix.	The folk name of each mushroom species being sold?	–
x.	Mushroom collected per season (kg)?	–
xi.	Usage of gathered mushrooms (food, medicine, or income)?	–
xii.	Learning ways of traditional knowledge about macro-fungi?	–
xiii.	Basic marketing channels of wild and cultivated edible mushrooms?	–
xiv.	Economic aspects of wild and cultivated edible mushrooms in the studied area?	–
xv.	Methods of processing and preservation of mushrooms (freezing, sun drying, or salting)?	–
Xvi.	Therapeutic uses of mushrooms in the traditional pharmacopeia of the region?	–
